# Microsurgical resection of a large petroclival meningioma via translabyrinthine approach combined with middle fossa craniotomy

**DOI:** 10.3171/2022.1.FOCVID21253

**Published:** 2022-04-01

**Authors:** Abdullah Keleş, Stephanie Alyse Armstrong, Sima Sayyahmelli, Mehmet Sabri Gürbüz, Mustafa Kemal Başkaya

**Affiliations:** Department of Neurological Surgery, University of Wisconsin–Madison School of Medicine and Public Health, Madison, Wisconsin

**Keywords:** combined posterior transpetrosal approach, facial nerve monitoring, petroclival meningioma, translabyrinthine approach

## Abstract

Petroclival meningiomas arise from the upper two-thirds of the clivus at the petroclival junction and are reached via various approaches. As petroclival meningiomas expand, they displace the brainstem and basilar artery toward the contralateral side. Because of their proximity to critical structures and deep skull base location, surgical treatment is challenging. Although several approaches have been introduced, their rationales vary. Herein, the authors demonstrate microsurgical resection of a large petroclival meningioma via a translabyrinthine approach combined with middle fossa craniotomy. For each approach, the pros and cons should be carefully evaluated based on the patient’s presentation and lesion characteristics.

The video can be found here: https://stream.cadmore.media/r10.3171/2022.1.FOCVID21253

## Transcript

In this video we present a microsurgical resection of a large petroclival meningioma via the translabyrinthine approach combined with middle fossa craniotomy. Petroclival meningiomas are a formidable challenge for surgeons with their deep, central skull base location and the presence of surrounding important neurovascular structures. While various approaches have been described, the rationale for the selection of each approach varies.

### 0:40 Case Presentation.

We present a case of a 68-year-old female patient with history of hyperlipidemia and a gastric ulcer disease. A year ago, she had a 30-minute episode of altered speech and mental fogginess. During an MRI workup for a possible TIA, a petroclival lesion was found incidentally. Recently, she developed severe constant headaches, difficulty with walking, and a mild swallowing difficulty, including coughing while eating. She was placed on a modified dysphagia diet. Her audiogram showed left-sided hearing loss with 60% speech discrimination. Otherwise, she was neurologically intact.

### 1:12 Preoperative Imaging.

MRI demonstrated a large homogenously enhancing petroclival mass with a dural tail, which is most consistent with meningioma. Preoperative imaging shows a hypoplastic right vertebral artery, mild narrowing and distortion of the left vertebral artery, contralateral displacement of the basilar artery, and compression of the brainstem along with hydrocephalus. Also, we can see tumor extension up to the dorsum sella and dural involvement of the left IAC.

### 1:36 Management.

Given the tumor size and location, the mass effect on the brainstem, the presence of obstructive hydrocephalus, and the patient’s symptoms, we recommended surgical resection. The patient elected to proceed with surgery. Since the patient’s hearing level was borderline and there was the likelihood of additional hearing loss during dissection of the tumor from the eighth cranial nerve, we chose a translabyrinthine approach over a retrolabyrinthine approach. The surgical goal was gross-total or near-total resection of the tumor without causing any disabling neurological deficits. Translabyrinthine approach was performed combined with middle fossa craniotomy, with continuous motor evoked potential and facial nerve monitoring.

### 2:10 Patient Positioning and Skin Incision.

The patient was positioned supine in a Mayfield head holder with her head turned 60°–70° to the right and slightly extended. A curvilinear incision was outlined, extending inferoposteriorly from the mastoid process, wrapping superiorly around the ear, and continuing into the middle fossa. Her head was then carefully prepped and draped. The incision was carried out. A large piece of temporalis fascia and muscle was harvested as a graft for later use in closure.

### 2:35 Temporal Bone Exposure.

Operative microscope was then brought into the field and the external acoustic canal, mastoid tip, and temporal line were all visualized. According to these landmarks, we delineated the borders, and the cortical mastoidectomy was then commenced. We then identified the antrum. The sigmoid sinus was skeletonized with the eggshell technique and reflected posteriorly. A middle fossa bony plate that extended from the superior petrosal sinus to the zygomatic root was removed. We then continued with the translabyrinthine approach. The facial nerve was skeletonized and left bony covered from the stylomastoid foramen. The posterior, superior, and lateral semicircular canals were identified and carefully removed. Then the vestibule and the cochlear aqueduct were opened. A dural cut was then made to enable cerebrospinal fluid aspiration to aid brain relaxation. Then the internal auditory canal was skeletonized circumferentially around 180°. The remaining bony cover over the superior petrosal sinus was removed. We then opened the dura of the internal acoustic canal and visualized the seventh and eighth cranial nerves. The inferior vestibular nerve, superior vestibular nerve, and facial nerve were all identified. The facial nerve was confirmed using stimulation. Here we can see a portion of the tumor that is medial and inferior to cranial nerves VII and VIII. In this part of the surgery, we took over the case from our neuro-otology colleagues who had performed the translabyrinthine portion of the surgery. At this stage, we performed a middle fossa craniotomy in addition to the part of the middle fossa plate which was drilled off during the translab approach.

### 3:55 Dural Opening.

We then opened the posterior fossa dura in the presigmoid region and middle fossa dura. The superior petrosal sinus was exposed, and clip ligation of the superior petrosal sinus was performed anterior to the drainage of the vein of Labbé. During preoperative imaging, it is critical to have studied anatomical variations of the drainage of the vein of Labbé. In this stage, care should be taken to preserve the drainage of the vein of Labbé. An incision was made in the tentorium toward the incisura, and the trochlear nerve was thus exposed on the midbrain surface. The tentorial cut provides wider supratentorial access to the tumor, which in this case made it easier to access the most upper portions of the tumor.

### 4:33 Tumor Exposure and Resection.

We now turned our attention back to dural opening. Here we can see the tumor and cranial nerves V, VII, and VIII. Before initiating the tumor resection, we performed facial nerve stimulation and assessed the relationship between the tumor and the cranial nerves. After identifying the surface anatomy of the tumor and adjacent neurovascular structures, we opened a window between the cranial nerves and began to enucleate the tumor. Ideally, the base of the tumor should be attacked first to interrupt its blood supply, which comes from the clival and petrosal dura, and the tentorium. However, this may not be possible with large petroclival meningiomas. With alternating internal debulking, hemostasis, and piecemeal tumor removal, we were able to shrink the tumor. As we dissected inferiorly to the seventh and eighth cranial nerves, the lower cranial nerves IX, X, XI were visualized. Further internal debulking allowed us to proceed with tumor detachment from the clival dura and to coagulate the feeding vessels. We then noticed erosion of the clivus by the tumor, which made hemostasis more difficult. With dural detachment, our objective was to achieve early devascularization because of the highly vascular lesion.

We then turned our attention to the inferior part of the tumor, which we carefully dissected from the ipsilateral vertebral artery. We proceeded to shrink the tumor through bipolar coagulation. This gave us enough space to visualize and free the basilar artery. Piecemeal resection was continued using microsurgical technique. Here we carefully dissected the tumor from the VII and VIII cranial nerve complex. Then we continued alternating hemostasis and piecemeal tumor removal. After sufficient tumor resection, we visualized the vertebral artery and origin of the posterior inferior cerebellar artery. Working between the cranial nerve VII and VIII complex and cranial nerve V, we encountered the anterior inferior cerebellar artery and partially dissected out it from the tumor. Then, the motor root of the trigeminal nerve came into view.

We turned our attention back to the dural base and continued with dural detachment of the tumor. During the detachment, we encountered profuse bleeding from the clival dura. We controlled this with bipolar coagulation, Gelfoam, and drilling using a coarse diamond bit without irrigation. Neurosurgeons must be aware of the tumor’s dural feeders and to be prepared for potential profuse bleeding from the dural base. After hemostasis was achieved, we continued the detachment of the tumor. We then focused on the inferior portion of the tumor. The most inferior portion was pulled upward for easier resection and removed in piecemeal fashion. The dissection was then proceeded superiorly, where we then encountered cranial nerve VI in Dorello’s canal. Using continuous sharp dissection, we completely freed cranial nerve VI from the tumor. Then we continued to dissect the anterior inferior cerebellar artery free from the tumor using sharp microsurgical techniques. With continuous dissection, we reached the posterior portion of the tumor and visualized the brainstem. Further dissection enabled us to expose the basilar artery and the origin of the anterior inferior cerebellar artery, which was completely freed from the tumor. We then removed the last piece of the tumor. We stimulated the facial nerve at 0.1 mA, which confirmed it was intact at the end of the surgery.

### 7:25 Closure.

We then opened the facial recess and removed the incus. The eustachian tube was filled tightly with rolled fascia and muscle tissue. The large temporalis fascia graft was placed over the translabyrinthine defect and carried superior onto the middle fossa defect. Previously harvested abdominal fat was cut into strips, and the posterior fossa was packed with these strips. The cranial bone was reaffixed over the middle fossa, and a multilayered closure of the subcutaneous layers and the skin was then performed.

### 7:51 Early Postoperative Course.

The patient woke up with a House-Brackmann grade 3 facial nerve weakness that progressed to grade 6 over 1 week. She also had temporary left fourth and sixth nerve palsies. Her preoperative dysphagia improved slightly, and her diet was upgraded prior to discharge. Histopathology of the tumor was confirmed as WHO grade I meningioma.

### 8:08 Follow-Up.

At 3-year follow-up, postoperative MRI confirmed gross-total resection of the tumor and no recurrence of the meningioma. It also demonstrated regression of the hydrocephalus. The patient’s double vision, walking difficulty, and headaches were completely resolved. Her facial nerve palsy improved from House-Brackmann grade 6 to grade 3.

### 8:25 Conclusions.

In conclusion, petroclival meningiomas arise from the dura along the upper two-thirds of the clivus at the petroclival junction. These can be approached via various supra- and infratentorial approaches.^1–3^ The rationale for approach selection varies according to the tumor size, location, extension, and patient presentation, including especially cranial nerve VII and VIII function and venous anatomy.^1,3,4^

Conventional supratentorial pterional and subtemporal approaches combined with anterior petrosectomy provide better visualization of the supratentorial compartments. Posterior petrosal approaches provide a more direct route to the base of the petroclival lesions than the supratentorial approaches. In large petroclival tumors with no serviceable hearing, the translabyrinthine approach combined with middle fossa craniotomy can be the first choice. This combined approach allows us to visualize the most upper portions of the tumor, which minimalizes postoperative cranial nerve deficits. It also allows for early tumor blood supply interruption.^1,3^ With the translabyrinthine approach combined with middle fossa craniotomy, we can have direct access to the most upper part of the clivus, the middle fossa, the cerebellopontine angle, and the anterolateral aspect of the brainstem.

Here you can see the selection criteria depends on the lesion location. Variations in venous anatomy, such as a high jugular bulb or alternative draining patterns of the vein of Labbé, may limit the effectiveness of the approach.^5^ Therefore, a careful preoperative evaluation of the venous anatomy is crucial for each case. Careful evaluation of the patient’s neurological findings, pathological anatomy, and hearing status is also essential. Small- to moderate-size meningiomas without significant supratentorial extension, and with a reasonable petroclival angle, can be safely removed with the retrosigmoid approach. In large petroclival meningiomas with nonserviceable hearing or borderline serviceable hearing with impaired BAERs, a translabyrinthine approach combined with middle fossa craniotomy may be used to achieve gross-total resection with minimal or no postoperative deficit.

## Acknowledgments

We thank Mark Pyle, MD, for his outstanding contribution to the translabyrinthine portion of the surgery.

## Disclosures

Dr. Başkaya reports being a consultant for Stryker.

## Author Contributions

Primary surgeon: Başkaya. Editing and drafting the video and abstract: Keleş, Armstrong, Sayyahmelli. Critically revising the work: Başkaya, Keleş, Armstrong, Sayyahmelli. Reviewed submitted version of the work: Başkaya, Keleş, Armstrong, Sayyahmelli. Approved the final version of the work on behalf of all authors: Başkaya. Supervision: Başkaya. Anatomic dissections: Gürbüz.

## References

[1] Coppens JR Couldwell WT Clival and petroclival meningiomas DeMonte F McDermott MW Al-Mefty O Al-Mefty’s Meningiomas Thieme; 2011 270 272

[2] Yaşargil MG Microneurosurgery. Georg Thieme Verlag 1996 134 165

[3] Erkmen K Pravdenkova S Al-Mefty O Surgical management of petroclival meningiomas: factors determining the choice of approach Neurosurg Focus 2005 19 2 E7 10.3171/foc.2005.19.2.8 16122216

[4] Xu F Karampelas I Megerian CA Selman WR Bambakidis NC Petroclival meningiomas: an update on surgical approaches, decision making, and treatment results Neurosurg Focus 2013 35 6 E11 10.3171/2013.9.FOCUS13319 24289119

[5] Avci E Dagtekin A Akture E Uluc K Baskaya MK Microsurgical anatomy of the vein of Labbé Surg Radiol Anat 2011 33 7 569 573 21279640 10.1007/s00276-011-0782-1

